# Custom AFM for X-ray beamlines: *in situ* biological investigations under physiological conditions

**DOI:** 10.1107/S1600577515016318

**Published:** 2015-09-30

**Authors:** B. Gumí-Audenis, F. Carlà, M. V. Vitorino, A. Panzarella, L. Porcar, M. Boilot, S. Guerber, P. Bernard, M. S. Rodrigues, F. Sanz, M. I. Giannotti, L. Costa

**Affiliations:** a ESRF, The European Synchrotron, Grenoble, France; b Institute for Bioengineering of Catalonia (IBEC), Barcelona, Spain; c Physical Chemistry Department, Universitat de Barcelona, Barcelona, Spain; d Networking Biomedical Research Center on Bioengineering, Biomaterials and Nanomedicine (CIBER-BBN), Madrid, Spain; e University of Lisboa, Falculty of Science, Biosystems and Integrative Sciences Institute – BIOISI, Lisbon, Portugal; f Institut Laue-Langevin, Grenoble, France; g ORTEC, Marseille, France; h CEA, LETI Grenoble, France

**Keywords:** *in situ* atomic force microscopy, grazing-incidence scattering and reflectivity, radiation damage, model lipid membranes

## Abstract

A fast atomic force microscope (AFM) has been developed that can be installed as a sample holder for grazing-incidence X-ray experiments at solid/gas or solid/liquid interfaces. It allows a wide range of possible investigations, including soft and biological samples under physiological conditions (hydrated specimens). The structural information obtained using the X-rays is combined with the data gathered with the AFM (morphology and mechanical properties), providing a unique characterization of the specimen and its dynamics *in situ* during an experiment. In this work, lipid monolayers and bilayers in air or liquid environment have been investigated by means of AFM, both with imaging and force spectroscopy, and X-ray reflectivity. In addition, this combination allows the radiation damage induced by the beam on the sample to be studied, as has been observed on DOPC and DPPC supported lipid bilayers under physiological conditions.

## Abstract 2

The performance of a custom atomic force microscope for grazing-incidence X-ray experiments on hydrated soft and biological samples is presented.

## 1. Introduction   

For several decades X-ray grazing-incidence techniques have been employed to characterize a large variety of samples, contributing significantly to extend our knowledge in many fields ranging from physics to biology and chemistry. Indeed, techniques such as X-ray reflectivity (XRR), grazing-incidence X-ray small-angle scattering (GISAXS) and grazing-incidence X-ray diffraction (GIXD) are powerful tools for characterizing surfaces at the nanoscale, providing structural information on the specimen in reciprocal space. In such types of experiments the information is averaged over the beam footprint on the specimen, which can range from hundreds of µm^2^ to several mm^2^ depending on the beam size and the experimental conditions. By providing structural information through the interaction between X-rays and the sample electronic structure, the techniques do not require any mechanical interaction with the specimen and therefore cannot evaluate its mechanical properties.

The atomic force microscope (AFM) is instead an instrument which characterizes surfaces at the nanoscale at the single molecule level in direct space and provides in addition information about its mechanical properties (Binnig *et al.*, 1986 ▸; Garcia & Perez, 2002 ▸; Butt *et al.*, 2005 ▸). However, the lateral resolution in-plane and the vertical resolution obtained by the AFM might be inferior to the X-ray techniques.

The combination of both techniques has already been reported and several operational schemes have already been introduced and successfully tested. First, a conductive AFM tip has been used as a local detector, either to collect the total yield (Rodrigues *et al.*, 2008 ▸; Rodrigues, 2009 ▸) or the XAFS–XEOL (X-ray absorption fine structure–X-ray excited optical luminescence) spectroscopy signal (Fauquet *et al.*, 2011 ▸) in grazing-incidence configuration, or to perform X-ray scanning transmission microscopy in normal incidence (Pilet *et al.*, 2012 ▸). A second line of experiments consists of the use of the AFM as a mechanical indenter of nano-sized systems while the X-ray beam is used to investigate the change of the specimen lattice parameter (Rodrigues *et al.*, 2009 ▸; Scheler *et al.*, 2009 ▸; Ren *et al.*, 2014 ▸), and related operational schemes such as that recently reported for an AFM–µLaue diffraction experiment (Leclere *et al.*, 2015 ▸). In all cases, apart from reference (Pilet *et al.*, 2012 ▸), the mechanical oscillator employed to perform tapping-mode AFM was piezoelectric (tuning fork or Akiyama probe) oscillating with high *Q*-factor but with high spring constant. This has limited the applications of the X-ray–AFM powerful combination to a very narrow range of experiments, preventing investigations in liquids on soft and biological materials where cantilevers with a stiffness inferior to 1 N m^−1^ have to be employed, as well as Electrostatic Force Microscopy (EFM) or Magnetic Force Microscopy (MFM) where a specific tip coating is needed.

We have built a fast AFM that can be installed as a synchrotron radiation sample holder (X-AFM). Our instrument aims to extend the capabilities of the X-ray–AFM combination to the previously mentioned scientific fields, allowing the use of any commercially available AFM cantilever. Despite the large use of the AFM as an *ex situ* characterization tool in X-ray oriented studies, scientists can profit from the *in situ* combination of the AFM and the X-ray grazing-incidence techniques from many points of view. At first, triggered sample changes such as phase transitions and chemical reactions can be investigated by both techniques simultaneously, whereas in the above-mentioned cases the AFM is conventionally employed off-line at the end of the X-ray experiment. Then, the AFM can be employed to align a nano-object with the X-ray beam and eventually exert forces such as mechanical, electric or magnetic on the studied structure. Finally, induced radiation damage of the specimen can be monitored in direct space in real time.

The instrument has been mounted and tested at the ID03 beamline of the ESRF. As a first test we have characterized monolayers of 1,2-distearoyl-*sn*-glycero-3-phosphoethanol­amine at the mica/air interface with the AFM while being irradiated by the X-ray beam to prove the compatibility of the instrument with the ID03 endstation. Then, model lipid membranes of 1,2-dipalmitoyl-*sn*-glycero-3-phosphocholine and 1,2-dioleoyl-*sn*-glycero-3-phosphocoline at the silicon/water interface have been characterized by XRR, AFM and force spectroscopy, providing a simultaneous study of the membranes from the nanoscale in reciprocal space to the mesoscale in direct space which can be correlated with their mechanical properties evaluated by means of force spectroscopy. As previously shown, the mechanical stability of a model lipid membrane is the fingerprint of that membrane with a defined lipid composition and under specific environmental conditions (Redondo-Morata *et al.*, 2012 ▸). In a third stage, we have investigated the effects of the radiation damage induced on the membranes with the AFM, observing an increase of the sample disorder, in particular the deposition of material on top of the membranes.

## 2. Instrument description   

**Figure 1:**
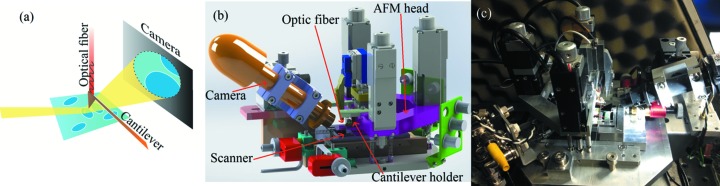
(*a*) Grazing-incidence experimental operational scheme in the beamline. (*b*) Overview of the X-AFM for the ESRF ID03 endstation. (*c*) Photograph of the AFM off-line.

The AFM has been designed to fulfill the specific needs of X-ray grazing-incidence experiments (Fig. 1*a* ▸):

(i) The weight of 1.350 kg is considerably lower than the existing commercial AFMs, allowing the installation of the instrument on top of several hexapodes.

(ii) The AFM is still operational if rotated in-plane and can be rotated out-of-plane by up to 10°. Rotations are necessary to align the sample and the beam under grazing-incidence conditions.

(iii) The setup permits the detection of the scattered beam out of the sample plane for reflectivity experiments and in-plane for surface scattering and diffraction experiments.

### 2.1. Overview   

The structure of the instrument is shown in Fig. 1(*b*) ▸. It has been designed to be a sample-scanning AFM and therefore the AFM tip is not displaced during the experiment. The AFM is composed of two separated parts: the microscope head and the microscope base; both are made of aluminium. When tilted, both the AFM head and base are tilted since they are kept together by six springs. In the microscope head, the AFM cantilever is mechanically clamped inside a cantilever holder. Two versions of this holder have been fabricated: one in aluminium and the other in Macor. The Macor version is employed when using conductive cantilevers in order to detect the photoelectrons generated in the tip by the incident X-ray beam. This allows the alignment between the AFM tip and X-ray beam with a single micro- or nano-structure when using very small beams. When alignment is not necessary, we employ the aluminium version that is more robust. An optical fiber is displaced in *X*/*Y*/*Z* over the cantilever back-side using a stage composed of three inertial motors (SLC Smaract). The microscope head approaches the microscope base with three long-range step-motors (8CMA06-13-15 Standa). The final tip–sample approach is then completed using the scanner mounted on the microscope base. The microscope base is on top of a table. Inside, a custom-made *X*/*Y*/*Z* scanner is installed. Samples are usually fixed either directly on top of the scanner, on top of the sample holder in aluminium covered with Teflon disks or on a Teflon sample holder in the case of measurements at the solid/liquid interface. In addition, a small Peltier element can be placed below the sample in order to induce temperature-dependent phase transitions of the specimen varying from 293 K to 320 K. Finally, an optical camera (Veho VMS-004) facilitates both the fiber–cantilever and tip–sample alignments.

### 2.2. Cantilever holder   

**Figure 2:**
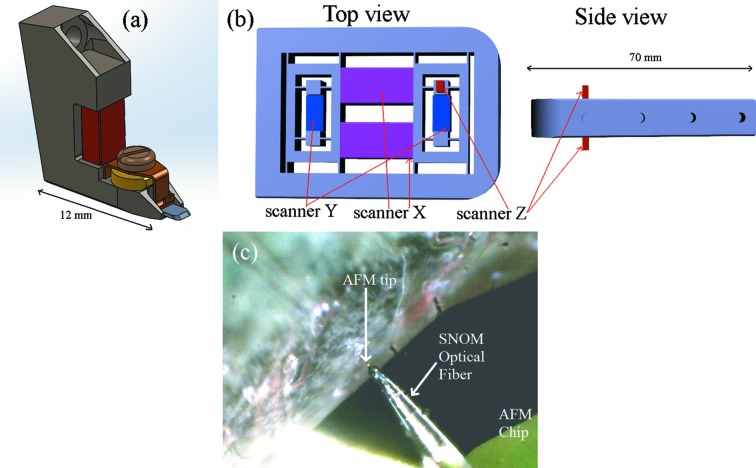
(*a*) Cantilever holder of the AFM. The red part shows the piezoelectric element that excites the cantilever at its resonance. (*b*) Top and side views of the AFM custom-made fast scanner. (*c*) Alignment of the high-speed cantilever with a SNOM optical fiber in proximity of a sample.

The cantilever holder is shown in Fig. 2(*a*) ▸. The AFM cantilever chip is mechanically clamped onto the holder with a screw. Any commercially available cantilever can be mounted on this instrument, permitting a large variety of possible experiments, ranging from the study of soft and biological materials in liquids (soft cantilevers) to MFM and EFM experiments (magnetic and conductive cantilevers). A piezoelectric element is employed to excite mechanically the cantilever at its resonance in order to acquire the specimen morphology in amplitude modulation mode (AM-AFM). The cantilever holder can be easily inserted and removed from the microscope head thanks to small magnets positioned both on the holder back-side and the AFM head. This facilitates exchange of the old AFM cantilever with a new one. In the case of MFM experiments, the magnets should be removed and the tip holder should be clamped mechanically to the microscope head.

### 2.3. Fabry–Perot interferometer   

The AFM tip position is measured with a fiber-optic-based interferometer (Rugar *et al.*, 1989 ▸). This detection operational scheme has already been employed for different experiments (Vitorino *et al.*, 2015 ▸; Costa *et al.*, 2014 ▸). A 125 µm-diameter cleaved optical fiber is brought to a distance of about 10 µm from the cantilever back-side by the inertial motors (SLC Smaract). The inertial motor which displaces the fiber perpendicularly to the cantilever is then controlled in its linear dynamic range using a proportional-integral controller in order to keep the tip–fiber distance constant in the low-frequency range (200 Hz). When working with very small cantilevers, necessary to achieve a fast imaging speed, a SNOM optical fiber has to be employed in the far-field regime. Indeed, a 125 µm-diameter fiber would not be able to come close enough to the small cantilever because of the presence of the AFM chip holding the cantilever (Fig. 2*c* ▸). The SNOM optical fibers are Lovalite pulled optical micro-tips with an apex radius of 100 nm. The laser employed is a 51nanoFI, whereas the coupler is a FBS-660-X, both from Shafter + Kirchhoff. The interference signal is converted into current by a photodetector and then converted into voltage with a FEMTO DHPCA-100 I/V converter.

### 2.4. Scanner   

The need of fast AFM imaging capabilities has important consequences on the design of the AFM for beamlines. As already mentioned, the use of a small cantilever resonating at high resonance is important. Then, a fast scanner and fast control electronics are needed. We have built a custom-made scanner which is sketched in Fig. 2(*b*) ▸. The structure is inspired by the scanner of the high-speed AFM (Ando, 2012 ▸) which allows the acquisition of several AFM images per second at solid/liquid interfaces. The main characteristic of such a mechanical structure is the insertion of second counter­balancing piezoelectric *X*, *Y* and *Z* elements as *X*, *Y* and *Z* scanners. In this way the center of mass of the structure is not displaced during scanning. This permits the bandwidth of the system to be increased, particularly for the *Z* scanner, allowing fast compensation for changes in the AFM tip oscillation amplitude in tapping-mode AFM. The scanner has been calibrated through the AFM characterization of standard calibration gratings. The scanning area is 12 µm (*X*) × 6 µm (*Y*) × 1 µm (*Z*). In order to acquire AFM images in different parts of the sample, the entire structure of the scanner can be displaced below the AFM tip using two picomotors (in red in Fig. 1*b* ▸).

### 2.5. Electronics   

The electronics driving the AFM are a complete Nanonis SPECS consisting of the modules RCS, SC4, OC4 and the power supply to drive the piezoelectric elements of the scanner. The AFM images and force curves are acquired using the software included with the electronics. Two additional custom-made *LabVIEW* programs drive the step motors that control the long-range approach between the AFM head and its base and the picomotors which displace the scanner.

## 3. Materials and methods   

### 3.1. Materials   

1,2-Distearoyl-*sn*-glycero-3-phosphoethanolamine (DSPE), 1,2-dipalmitoyl-*sn*-glycero-3-phosphocholine (DPPC) and 1,2-dioleoyl-*sn*-glycero-3-phosphocoline (DOPC) were purchased from Sigma-Aldrich (St Louis, MO, USA). The experiments with DSPE monolayers were carried out under ambient conditions (at room temperature and at the solid/air interface). DPPC and DOPC bilayers experiments were performed in buffer solution of 150 m*M* NaCl, 20 m*M* MgCl_2_, 20 m*M* Hepes (pH 7.4) prepared with ultrapure water (Milli-Q reverse osmosis system, 18.2 mΩ cm resistivity) and filtered before use with an inorganic membrane filter (0.1 µm pore size).

### 3.2. Sample preparation   

#### 3.2.1. DSPE monolayers   

DSPE was dissolved in chloroform to give a final concentration of 0.13 m*M*. Afterwards, 10 µL were deposited onto freshly cleaved mica substrate with dimensions of 4 mm × 4 mm × 0.1 mm and left to dry under ambient conditions. The mica substrates were cut from mica disks, 14 mm diameter, purchased from Agar Scientific.

#### 3.2.2. DPPC and DOPC bilayers   

DPPC and DOPC were individually dissolved in chloroform:methanol (3:1) to give a final concentration of 3 m*M*. Afterwards, the solvent was evaporated to dryness under nitrogen flow in order to achieve a thin film on the walls of the tube. Then, the dried phospholipid films were hydrated with buffer solution, previously heated above the transition temperature (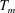) of the lipid, until a final total concentration of 0.5 m*M*. The following step was to subject the falcon tubes to cycles of vortex mixing (1 min) and heating (20 s) to *ca.* 323 K. The vesicles suspensions were placed in an ultrasound bath for 30 min to finally obtain unilamellar vesicles (Attwood *et al.*, 2013 ▸; Mingeot-Leclercq *et al.*, 2008 ▸). Silicon wafers with orientation (100) ± 5°, polished (r.m.s. < 0.5 nm), 275 µm thickness and 50.8 mm diameter were purchased from CrysTec. Square substrates with dimensions 4 mm × 4 mm were obtained from the wafers and subsequently exposed to plasma (Plasma Cleaner, Harrick-Plasma) at high RF power level for 5 min in order to activate the hydrophilic bonding of the Si surfaces. The supported lipid bilayers (SLBs) were obtained by depositing 20 µL of the suspension of unilamellar vesicles onto the hydrophilic silicon substrate for 10 min at a temperature above 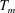 of the lipid. Then, the samples were rinsed with buffer solution in order to remove the vesicles not fused, always keeping the silicon substrate hydrated.

### 3.3. AFM imaging   

AFM images were performed with the fast AFM described above. All the images were acquired in AFM amplitude-modulation mode in a large oscillation amplitude regime: the free oscillation amplitude imposed to the tip ranged from 10 to 40 nm depending on the experiment.

#### 3.3.1. DSPE monolayers   

The acquired AFM images were obtained at room temperature and in air. We used AC55-Olympus cantilevers with nominal spring constant of 85 N m^−1^ resonating at 2.15 MHz and a SNOM optical fiber as Fabry–Perot plate. The same scanned area (3 µm × 3 µm) was imaged at different speeds, from 26 s per image to 1 s per image. In the experiments where the X-ray beam was irradiating the sample, the AFM images were acquired with an NSC15-µmasch cantilever, resonating at approximately 330 kHz, at a speed of 30 s per image. We used NSC15 cantilevers, slower than and twice as soft as the AC55 cantilevers, to facilitate the acquisition of the AFM images due to the higher level of vibrations present on the diffractometer compared with the off-line configuration.

#### 3.3.2. DPPC and DOPC bilayers   

All the images were acquired at room temperature and under buffer conditions 150 m*M* NaCl, 20 m*M* MgCl_2_, 20 m*M* Hepes (pH 7.4). We used V-shaped Si_3_N_4_ cantilevers with a nominal spring constant of 0.35 N m^−1^ (SNL, Bruker AFM Probes; Camarillo, CA, USA) and an optical fiber as Fabry–Perot plate. The scanned region was 12 µm × 6 µm and the scan speed was in the range 1 min per image to 8 min per image.

### 3.4. AFM-based force spectroscopy (AFM-FS)   

AFM-FS has emerged as an excellent tool for characterizing the mechanical properties of SLBs, as it is especially valuable in terms of spatial accuracy and force resolution (Redondo-Morata *et al.*, 2012 ▸). The maximum force the bilayer is able to withstand before breaking (breakthrough force, *F*_b_) is directly related to the lateral interactions between lipid molecules (Dufrene *et al.*, 1998 ▸) and it is affected by variables like temperature, lipid chemical composition, pH, ionic strength and electrolyte composition (Garcia-Manyes & Sanz, 2010 ▸; Garcia-Manyes *et al.*, 2010 ▸; Redondo-Morata *et al.*, 2014 ▸). As a consequence, *F*_b_ clearly fingerprints the nanomechanical stability of SLBs of defined composition and in a specific environment. In AFM-FS experiments, *F*_b_ is observed as a discontinuity on the approach force–distance curve when the AFM tip breaks through to the SLB. After imaging a region of interest characterized by large SLB areas, deflection–*Z* piezo displacement curves (easily converted into force–distance curves by calibrating the cantilever spring constant) were recorded by approaching and retracting the cantilever tip to the sample at constant velocity (300 nm s^−1^). We acquired the force–distance curves by defining a grid on a previously scanned region and performing a curve in each point using V-shaped Si_3_N_4_ cantilevers with nominal spring constant of 0.35 N m^−1^ (SNL, Bruker AFM Probes, Camarillo, CA, USA) and with an optical fiber as Fabry–Perot plate.

### 3.5. X-ray reflectivity   

We used high-energy X-rays, 22.5 keV, corresponding to λ = 0.551 Å. The use of high energy was required to penetrate through the buffer layers (Miller *et al.*, 2005 ▸) more efficiently than conventional measurements at 10 keV. The beam size was 300 µm × 30 µm (horizontal × vertical). The flux at the sample position was 2 × 10^13^ photons s^−1^. A set of attenuators was used in order to control the incident photon flux and limit the exposure of the sample to X-rays. Indeed, beam damage was observed when the sample was illuminated with the full beam after a few minutes of exposure; for this reason the data acquisition was limited to *Q* = 0.6 Å^−1^. At these values the signal is still visible using a filter with a transmission of 0.067. A plot reporting the transmitted beam after the filters used during the measurement is given in the supporting information (Fig. S1). Each point in the reflectivity curve was measured limiting the exposure to 1 s. Each reflectivity curve was acquired in 20 min. We acquired 209 points that correspond to a total exposure time of 3 min and 29 s and a dose of approximately 23 × 10^3^ Gy, calculated from Elleaume *et al.* (1999 ▸). Data have been treated modeling the interface with a seven-slabs model (Novakova *et al.*, 2006 ▸; Daillant *et al.*, 2005 ▸) including a silicon oxide layer of 1 nm, a layer of water molecules in between the substrate and the lipid heads, two hydrocarbon tails regions including a CH_3_ slab in between and lastly the second lipid heads slab. Data have been fitted employing the *GenX* software.

## 4. Results   

### 4.1. AFM imaging at the solid/air interface   

**Figure 3:**
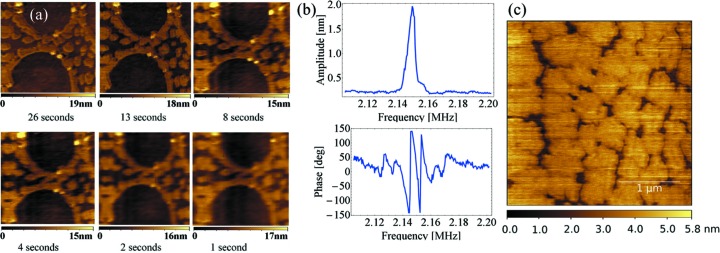
(*a*) Repeated images of DSPE monolayers deposited onto mica at different acquisition speeds. The highest speed is 1 s per image which has lower resolution than the images obtained slowly but yet acceptable resolution since the morphological details are still observed. Scanned area: 3 µm × 3 µm. (*b*) Transfer function of the fast AC55-Olympus cantilever. (*c*) DSPE monolayers deposited onto mica imaged with the AFM mounted on the diffractometer of ID03. The quality of the image is close to the images obtained off-line (*a*).

At first the fast AFM was tested off-line. We imaged DSPE phospholipid monolayers deposited onto mica under environment conditions. The same scanning area was imaged at different speeds. It is possible to acquire images at 1 s per image keeping an acceptable lateral and vertical resolution compared with images acquired slowly, usually in 30 s (Fig. 3*a* ▸). Fig. 3(*b*) ▸ shows the cantilever transfer function once it is mechanically excited. The cantilever resonance frequency is higher than the resonance of the small piezoelectric element clamped in the cantilever holder: this results in a non-linear excitation as a function of the frequency and consequently in higher phase-shift at resonance.

Then, the AFM was mounted on the hexapode of the first hutch of the ESRF ID03 endstation. The higher level of vibration of the diffractometer did not permit the acquisition speed of the order of 1 s per image to be reached. However, it has been possible to acquire images of the DSPE monolayers with a vertical resolution inferior to 1 nm with a quality comparable with the images obtained *ex situ*. Fig. 3(*c*) ▸ shows an image acquired at 30 s per image during this experimental session.

### 4.2. AFM imaging and reflectivity at the solid/liquid interface at ID03   

**Figure 4:**
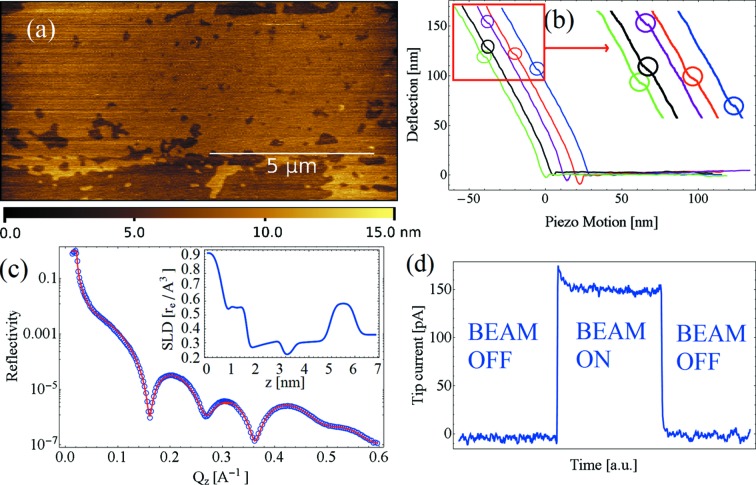
(*a*) AFM image of DPPC bilayers deposited onto silicon under physiological conditions. (*b*) Several force spectroscopy curves acquired with the AFM showing the deflection of the cantilever as a function of the sample displacement (piezo motion): we observed the rupture of the bilayers once a vertical deflection of the cantilever in the range 100–150 nm is measured. The curves have been shifted by 6 nm in the *X*-axis for better clarity. (*c*) Associated XRR from which we evaluate the bilayer thickness of 5.5 nm. Experimental data (blue circles) and best fit (continuous red line). Inset: scattering length density profile evaluated from the best fit of the reflectivity data. (*d*) Current flowing in the Si_3_N_4_ cantilever once aligned with the X-ray beam.

In a second experimental session we characterized DPPC supported bilayers deposited onto silicon under physiological conditions with AFM and XRR simultaneously. Fig. 4 ▸ shows an AFM image and the associated XRR and force spectroscopy curves enlightening the necessary force applied by the AFM tip to break the bilayers. In order to align the AFM tip with the X-ray beam and the bilayers, we measured the current flowing in the AFM cantilever once irradiated by the beam. Since the size of the beam is here comparable with the size of the cantilever, we assume the entire cantilever and the tip to be aligned with the beam. Fig. 4(*d*) ▸ presents the measurement of the current once the X-ray beam is turned on. We have usually observed a few hundreds of picoamperes for these cantilevers and the X-ray intensity available at ID03. In addition, we have developed an alternative protocol employing scanning X-ray transmission microscopy to align the beam with the AFM cantilever (see Fig. S2 of the supporting information).

The reflectivity curve presented in Fig. 4(*c*) ▸ suggests a bilayer thickness of 5.5 nm. The morphological study of the AFM image reveals a bilayer thickness of 5.1 nm which is lower than the thickness provided by the reflectivity data. We explain the difference in thickness as being due to membrane compression during acquisition of the AFM image in amplitude modulation mode. Fig. 4(*b*) ▸ shows the cantilever deflection–piezo motion curves registered for the membranes once a vertical force is exerted with the tip. Curves are measured in different locations on the specimen upon the definition of a 20 × 20 pixels grid; therefore curves are measured in locations which are roughly 500 nm distant. Considering the cantilever nominal spring constant, we evaluate the rupture of the membranes in the range 35–45 nN, as a clear discontinuity in the indentation part of the approach curve. Values typically found for DPPC SLBs are usually in the 20 nN range (Gumí-Audenis *et al.*, 2015 ▸). The deviation from typical rupture forces may be attributed to the use of the cantilever nominal spring constant, which was not calibrated in this experiment and can differ consistently from the real spring constant. These measurements are essential for further investigations to evaluate the effect of the radiation damage *in situ* on the mechanical properties of soft and biological samples. The best fit parameters for the reflectivity data are reported in §3 of the supporting information.

### 4.3. Observation of radiation damage of phospholipids at ID03   

**Figure 5:**
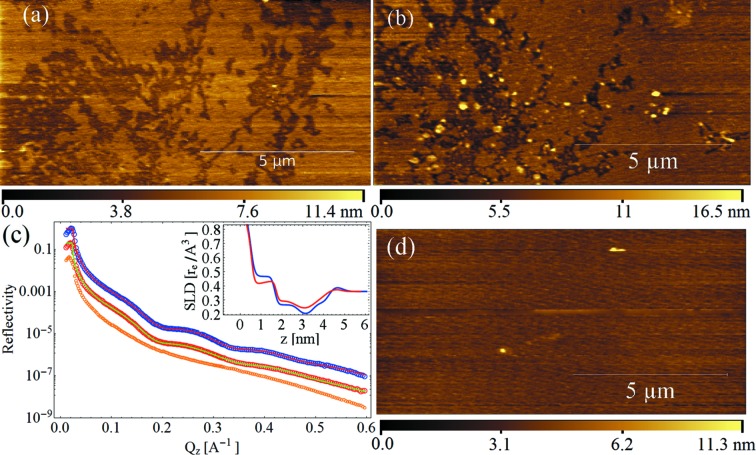
(*a*) AFM image of DOPC bilayers before being exposed to X-rays. (*b*) AFM image of DOPC bilayers damaged by the X-ray beam during the acquisition of the first reflectivity curve which has induced the deposition of material on top of the membranes. (*c*) Reflectivity curves. Blue and red: experimental data and best fit, respectively, for the data acquired after the AFM image shown in (*a*). Red and green (shifted for better clarity): experimental data and best fit, respectively, for the data acquired after the AFM image shown in (*b*). Orange (shifted for better clarity): experimental data for the data acquired after exposition of 5 min full beam without attenuation at an incident angle of 0.1°: the membranes have been removed by the beam and solely the substrate is observed. Inset: scattering length density profile evaluated from the fit. Blue: data acquired after the AFM image shown in (*a*). Red: data acquired after the AFM image shown in (*b*). (*d*) AFM image acquired after exposure to full beam without attenuation. The membranes are not observed by AFM in agreement with the data shown in orange in (*c*).

**Figure 6:**
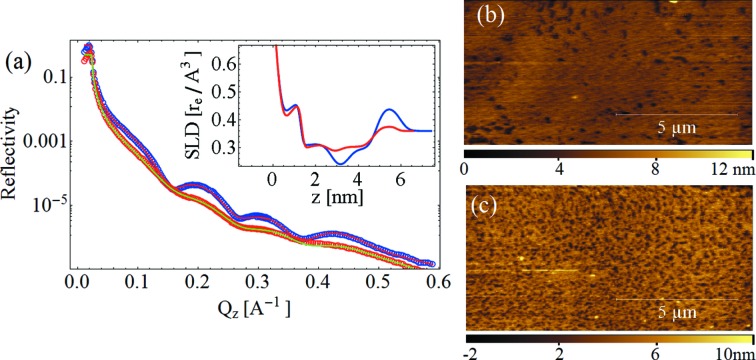
(*a*) Reflectivity curves on DPPC bilayers. Blue and red: experimental data and best fit, respectively, for the data acquired after the AFM image shown in (*b*). Red and green (shifted for better clarity): experimental data and best fit, respectively, for the data acquired after the AFM image shown in (*c*). Inset: scattering length density profile evaluated from the fit. Blue: data acquired after the AFM image shown in (*b*). Red: data acquired after the AFM image shown in (*c*). (*b*) AFM image of DPPC bilayers before being exposed to X-rays. (*c*) AFM image of DPPC bilayers damaged by the X-ray beam during the acquisition of the first reflectivity curve. We observe the formation of holes in the membrane.

As a final experiment, we monitored the radiation damage induced by the beam, first on DOPC and then on DPPC supported bilayers during the acquisition of a single reflectivity curve. In addition, DOPC bilayers were investigated after a long exposure in the absence of attenuation filters. We acquired both X-ray reflectivity and AFM images before and after the beam exposure in the very same place on the sample. The AFM images of Figs. 5(*a*), 5(*b*) and 5(*d*) ▸ show the changes induced on DOPC bilayers by the X-rays, whereas Fig. 5(*c*) ▸ presents the associated reflectivity curves. Fig. 6 ▸ shows the experiment performed on DPPC bilayers.

In both experiments a decrease in intensity in the reflectivity curves leading to the complete disappearance of the fringes is observed. Nevertheless, the AFM images show a completely different behavior in the two samples.

In the case of DOPC, we clearly observe deposition of material on top of the membranes: this can be easily noticed by comparing the AFM images presented in Figs. 5(*a*) and 5(*b*) ▸. Fig. 5(*c*) ▸ shows the reflectivity curves acquired after the AFM experiment presented in Fig. 5(*a*) ▸ (blue and red data) and after the AFM experiment presented in Fig. 5(*b*) ▸ (red and green data), the second being shifted for better clarity. Despite the similarity between the reflectivity curves, the AFM images clearly show morphological differences since aggregates are observed on top of the membranes. It is worth noticing that a change in the scattering length density (SLD) profile is also observed, which leads to the conclusion that the density of the bilayer at the nanometric scale has also been modified, suggesting a decrease of the membrane coverage. After exposition to 5 min full beam without attenuation at an incident angle of 0.1°, the membranes have totally disappeared, as shown by the orange reflectivity curve presented in Fig. 5(*c*) ▸ and the AFM image in Fig. 5(*d*) ▸. The best fit parameters for the reflectivity data are reported in §4 of the supporting information.

In the DPPC bilayers the formation of holes in the bilayer stucture at the micrometric and nanometric scale is clearly visible (Fig. 6*c* ▸) which is reflected in the XRR with an increase of roughness (Fig. 6*a* ▸). As in the case of DOPC, a change after irradiation of the SLD profile is observed, suggesting a decrease of the membrane coverage at the nanoscale.

The reason why the two systems show such different damage behavior cannot be explained on the mere basis of the data collected during the experiment. While the XRR data show a similar damage trend for both DPPC and DOPC [Figs. 5(*c*) ▸ and 6(*a*) ▸] leading to a decrease of the substrate coverage, the *in situ* AFM data suggest that the holes formation is occurring at the mesoscale in the case of DPPC while it is not observed on DOPC [Figs. 5(*b*) ▸ and 6(*c*) ▸]. Concluding, it is worth mentioning that we have observed radiation damage solely in the areas exposed to the beam.

## 5. Conclusion   

We have developed a fast AFM which can be integrated as a sample holder in X-ray beamlines allowing grazing-incidence X-ray experiments simultaneously with AFM topography and mechanical characterization at the nanoscale. The instrument has been successfully tested on the ID03 endstation of the ESRF both at the solid/air and solid/liquid interfaces. Model lipid membranes deposited onto silicon substrate have been characterized with both XRR and AFM. The structural information of the membrane, their morphological features in direct space and their nano-mechanics have been characterized simultaneously *in situ*. This shows that our instrument can be used to associate the structure of soft and biological samples with their mechanical characterization and morphology. In addition, the X-AFM can monitor radiation damage on soft and biological materials investigated by grazing-incidence X-ray techniques. Indeed, radiation damage effects have been detected by both AFM and XRR. Generally, any dynamic process requiring *in situ* characterization of the specimen to facilitate the X-ray experiment and data treatment, *i.e.* insertion of peptides or small molecules in model membranes or temperature-induced phase transitions, can be monitored by the AFM.

In addition, we are convinced that the *in situ* AFM–X-rays combination will be useful with nano-beams with a broad range of possible applications, ranging from spectroscopy and fluorescence to scattering from single molecules mechanically deformed by a controlled external force exerted by the AFM tip. The combination will become even more powerful with the on-going progress in X-ray free-electron laser technology.

Other developments in progress include the use of large colloidal probes with diameters of the order of 50–100 µm in order to employ the vertical pressure exerted by the cantilever on soft thin films and correlate the structural change observed by the X-rays with the induced exerted force. This operational scheme would permit a structural study of confined molecules and atoms at the nanoscale which has a broad range of investigations as demonstrated by surface force apparatus studies (Israelachvili, 2011 ▸): an example would be the mechanism of vesicles fusion (Abdulreda & Moy, 2007 ▸).

## 6. Access to the instrument   

Access to the AFM for beamlines is open to all ESRF users. Users are encouraged to contact the Surface Science Laboratory staff to organize training periods and discuss the experiment and opportunities for collaborations. Depending on the users’ scientific needs, two additional AFMs for beamlines might be employed: one for grazing-incidence experiments with larger scanning area (Costa *et al.*, 2014 ▸) and one to couple fast AFM with X-ray techniques in normal-incidence configuration (in commissioning).

## Supplementary Material

1: Filter setting used during the acquisition of the Reflectivity data; 2: Beam-tip alignment; 3: Reflectivity for DPPC; 4: Reflectivity for DOPC.. DOI: 10.1107/S1600577515016318/fv5037sup1.pdf
