# Supplementary material for: Custom AFM for X-ray beamlines: in situ biological investigations under physiological conditions
Source: J Synchrotron Radiat. 2015 Sep 30;22(Pt 6):1364–71. doi: 10.1107/S1600577515016318 (PMC4787838; doi:10.1107/S1600577515016318)
Supplement: Supplementary file 1 [file s-22-01364-sup1.pdf]

JOURNAL OF SYNCHROTRON RADIATION

Volume 22 (2015)

Supporting information for article:

Custom AFM for X-ray beamlines: *in situ* biological investigations under physiological conditions

B. Gumí-Audenis, F. Carlà, M. V. Vitorino, A. Panzarella, L. Porcar, M. Boilot, S. Guerber, P. Bernard, M. S. Rodrigues, F. Sanz, M. I. Giannotti and L. Costa

S1- Filter setting used during the acquisition of the Reflectivity data.

Figure S1 shows the transmitted beam after the filter as a function of Q_z employed for the acquisition of a Reflectivity curve. The flux at the sample position without filters was 2 10^{13} photons/s.

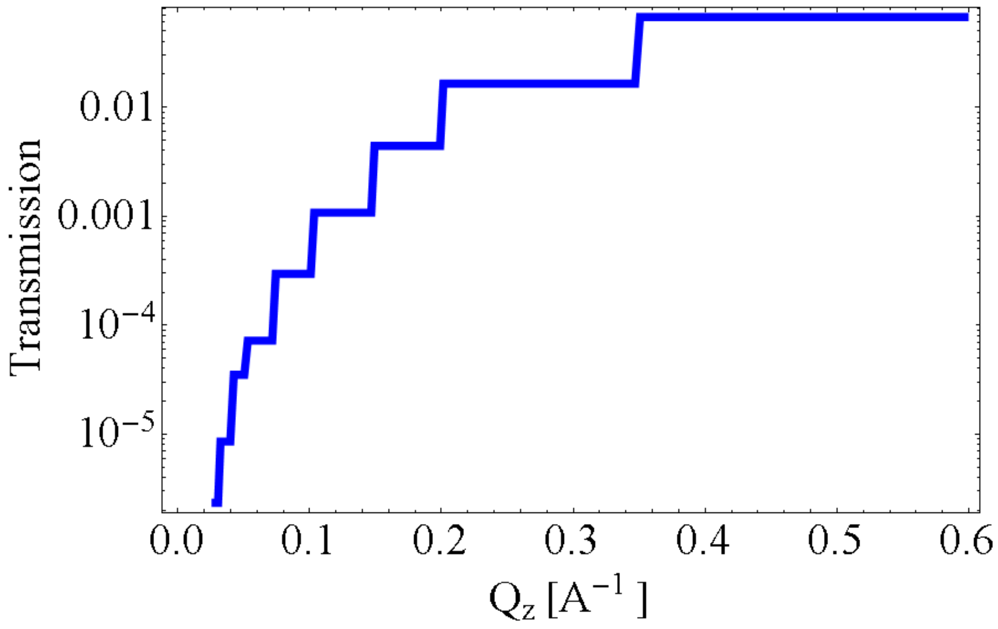

Figure S1: Transmitted beam used for the acquisition of the Reflectivity curves as a function of Q_z.

S2- Beam-Tip alignment:
The AFM cantilever and the beam have been aligned either by measuring the current flowing in the cantilever (see Figure 4d in the main manuscript) or by Scanning X-Ray Transmission Microscopy. Figure S2 shows the transmitted beam scans measured at different heights with the ID03 detector. The inset presents schematically the scan geometry. Black curve: the beam crossing the optical fiber and subsequently the AFM chip. Red curve: beam crossing solely the optical fiber. Blue curve: beam crossing the AFM cantilever.

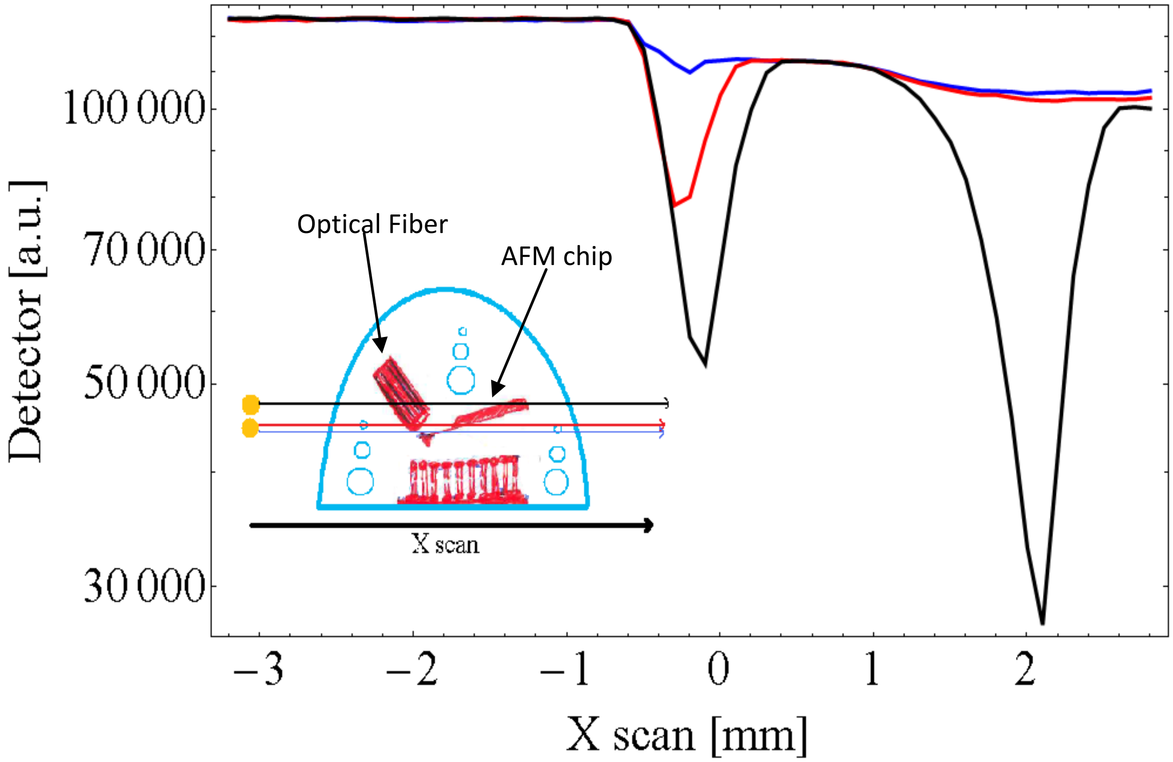

Figure S2: transmitted beam scans measured along the X-Axes at different heights corresponding to different colours. Inset: pictorial scheme of the measurement set up.

S3- Reflectivity for DPPC: Best fit parameters for data shown in Figure 4c of the main manuscript:

Derived values for thicknesses (d) , electron density (ρ) and the roughness parameter (σ) of the first head-groups H_1 (the closest to the substrate), the two hydrocarbon chains C_1 and C_2, the CH_3 group and the second head-groups H_2 from the best fit of the data presented in Figure 4c.
σ_{H20} , σ_{SiO2} and σ_{Si} are the roughness parameters for the water layer below the head-groups H_1, the SiO_2 and the Silicon Substrate.

**Parameter**	**DPPC**
d_{H1} (nm)	0.76
d_{C1} (nm)	1.55
d_{CH3} (nm)	0.49
d_{C2} (nm)	1.60
d_{H2} (nm)	1.09
ρ_{H1} (e^-/A^3)	0.58
ρ_{C1} (e^-/A^3)	0.31
ρ_{CH3} (e^-/A^3)	0.23

ρ_{C2} (e^-/A^3)	0.31
ρ_{H2} (e^-/A^3)	0.55
σ_{H1} (at/A)	1.00
σ_{C1} (at/A)	1.00
σ_{CH3} (at/A)	1.34
σ_{C2} (at/A)	1.89
σ_{H2} (at/A)	1.82
σ_{H20} (at/A)	1.00
σ_{SiO2} (at/A)	2.76
σ_{Si} (at/A)	23

S4 Reflectivity for DOPC: Best fit parameters for data shown in Figure 5c of the main manuscript:

Derived values for thicknesses (d) , electron density (ρ) and the roughness parameter (σ) of the first head-groups H_1 (the closest to the substrate), the two hydrocarbon chains C_1 and C_2, the CH_3 group and the second head-groups H_2 from the best fit of the data presented in Figure 5c.
σ_{H20} , σ_{SiO2} and σ_{Si} are the roughness parameters for the water layer below the head-groups H_1, the SiO_2 and the Silicon Substrate.

**Parameter**	**DOPC (blue data)**	**DOPC (red data)**
d_{H1} (nm)	1.12	1.18
d_{C1} (nm)	1.11	0.92
d_{CH3} (nm)	0.72	0.85
d_{C2} (nm)	0.75	0.59
d_{H2} (nm)	0.67	0.74
ρ_{H1} (e^-/A^3)	0.48	0.43
ρ_{C1} (e^-/A^3)	0.26	0.30
ρ_{CH3} (e^-/A^3)	0.21	0.27
ρ_{C2} (e^-/A^3)	0.28	0.35
ρ_{H2} (e^-/A^3)	0.40	0.38
σ_{H1} (at/A)	1.24	1.01
σ_{C1} (at/A)	2.17	2.99
σ_{CH3} (at/A)	2.19	2.82
σ_{C2} (at/A)	2.05	2.84
σ_{H2} (at/A)	2.27	1.94
σ_{H20} (at/A)	1.01	1.01
σ_{SiO2} (at/A)	2.84	2.84
σ_{Si} (at/A)	9.36	9.36
